# Radiomics preoperative-Fistula Risk Score (RAD-FRS) for pancreatoduodenectomy: development and external validation

**DOI:** 10.1093/bjsopen/zrad100

**Published:** 2023-10-09

**Authors:** Erik W Ingwersen, Jacqueline I Bereska, Alberto Balduzzi, Boris V Janssen, Marc G Besselink, Geert Kazemier, Giovanni Marchegiani, Giuseppe Malleo, Henk A Marquering, C Yung Nio, Riccardo de Robertis, Roberto Salvia, Ewout W Steyerberg, Jaap Stoker, Femke Struik, Inez M Verpalen, Freek Daams, for the Pancreatobiliary and Hepatic Artificial Intelligence Research (PHAIR) consortium

**Affiliations:** Department of Surgery, Amsterdam UMC, location Vrije Universiteit Amsterdam, Amsterdam, The Netherlands; Department of Surgery, Cancer Center Amsterdam, Amsterdam, The Netherlands; Department of Surgery, Amsterdam Gastroenterology Endocrinology and Metabolism, Amsterdam, The Netherlands; Department of Surgery, Cancer Center Amsterdam, Amsterdam, The Netherlands; Department of Radiology and Nuclear Medicine, Amsterdam UMC, location University of Amsterdam, Amsterdam, The Netherlands; Department of Biomedical Engineering and Physics Department, University of Amsterdam, Amsterdam, The Netherlands; Department of Surgery and Oncology, Unit of General and Pancreatic Surgery, University of Verona Hospital Trust, Verona, Italy; Department of Surgery, Cancer Center Amsterdam, Amsterdam, The Netherlands; Department of Surgery, Amsterdam Gastroenterology Endocrinology and Metabolism, Amsterdam, The Netherlands; Department of Surgery, Amsterdam UMC, location University of Amsterdam, Amsterdam, The Netherlands; Department of Surgery, Cancer Center Amsterdam, Amsterdam, The Netherlands; Department of Surgery, Amsterdam Gastroenterology Endocrinology and Metabolism, Amsterdam, The Netherlands; Department of Surgery, Amsterdam UMC, location University of Amsterdam, Amsterdam, The Netherlands; Department of Surgery, Amsterdam UMC, location Vrije Universiteit Amsterdam, Amsterdam, The Netherlands; Department of Surgery, Cancer Center Amsterdam, Amsterdam, The Netherlands; Department of Surgery and Oncology, Unit of General and Pancreatic Surgery, University of Verona Hospital Trust, Verona, Italy; Department of Surgery and Oncology, Unit of General and Pancreatic Surgery, University of Verona Hospital Trust, Verona, Italy; Department of Radiology and Nuclear Medicine, Amsterdam UMC, location University of Amsterdam, Amsterdam, The Netherlands; Department of Biomedical Engineering and Physics Department, University of Amsterdam, Amsterdam, The Netherlands; Department of Radiology and Nuclear Medicine, Amsterdam UMC, location University of Amsterdam, Amsterdam, The Netherlands; Department of Radiology, University Hospital G.B. Rossi, University of Verona, Verona, Italy; Department of Surgery and Oncology, Unit of General and Pancreatic Surgery, University of Verona Hospital Trust, Verona, Italy; Department of Biomedical Data Sciences, Leiden University Medical Center, Leiden, The Netherlands; Department of Surgery, Cancer Center Amsterdam, Amsterdam, The Netherlands; Department of Surgery, Amsterdam Gastroenterology Endocrinology and Metabolism, Amsterdam, The Netherlands; Department of Radiology and Nuclear Medicine, Amsterdam UMC, location University of Amsterdam, Amsterdam, The Netherlands; Department of Radiology and Nuclear Medicine, Amsterdam UMC, location University of Amsterdam, Amsterdam, The Netherlands; Department of Radiology and Nuclear Medicine, Amsterdam UMC, location University of Amsterdam, Amsterdam, The Netherlands; Department of Surgery, Amsterdam UMC, location Vrije Universiteit Amsterdam, Amsterdam, The Netherlands; Department of Surgery, Cancer Center Amsterdam, Amsterdam, The Netherlands

## Abstract

**Background:**

Accurately predicting the risk of clinically relevant postoperative pancreatic fistula after pancreatoduodenectomy before surgery may assist surgeons in making more informed treatment decisions and improved patient counselling. The aim was to evaluate the predictive accuracy of a radiomics-based preoperative-Fistula Risk Score (RAD-FRS) for clinically relevant postoperative pancreatic fistula.

**Methods:**

Radiomic features were derived from preoperative CT scans from adult patients after pancreatoduodenectomy at a single centre in the Netherlands (Amsterdam, 2013–2018) to develop the radiomics-based preoperative-Fistula Risk Score. Extracted radiomic features were analysed with four machine learning classifiers. The model was externally validated in a single centre in Italy (Verona, 2020–2021). The radiomics-based preoperative-Fistula Risk Score was compared with the Fistula Risk Score and the updated alternative Fistula Risk Score.

**Results:**

Overall, 359 patients underwent a pancreatoduodenectomy, of whom 89 (25 per cent) developed a clinically relevant postoperative pancreatic fistula. The radiomics-based preoperative-Fistula Risk Score model was developed using CT scans of 118 patients, of which three radiomic features were included in the random forest model, and externally validated in 57 patients. The model performed well with an area under the curve of 0.90 (95 per cent c.i. 0.71 to 0.99) and 0.81 (95 per cent c.i. 0.69 to 0.92) in the Amsterdam test set and Verona data set respectively. The radiomics-based preoperative-Fistula Risk Score performed similarly to the Fistula Risk Score (area under the curve 0.79) and updated alternative Fistula Risk Score (area under the curve 0.79).

**Conclusion:**

The radiomics-based preoperative-Fistula Risk Score, which uses only preoperative CT features, is a new and promising radiomics-based score that has the potential to be integrated with hospital CT report systems and improve patient counselling before surgery. The model with underlying code is readily available via www.pancreascalculator.com and www.github.com/PHAIR-Consortium/POPF-predictor.

## Teaser

Accurately predicting clinically relevant postoperative pancreatic fistula before surgery may assist surgeons in better counselling patients. The radiomics preoperative-Fistula Risk Score was generated using computed tomography features and shown to accurately predict postoperative pancreatic fistula. After further validation it has the potential to be integrated in hospital CT report systems.

## Introduction

Clinically relevant postoperative pancreatic fistula (CR-POPF) is a feared complication following pancreatoduodenectomy that negatively impacts short- and long-term outcomes^1,2^. Accurate risk stratification of CR-POPF in the preoperative setting can assist in determining the best surgical approach for high-risk or frail patients. In the perioperative interval, high-risk patients for CR-POPF may be candidates for prophylactic treatment such as somatostatin analogues^3^. In patients with cystic lesions of the pancreatic head, accurate risk stratification of CR-POPF can help decide whether to proceed with a pancreatoduodenectomy or consider alternative approaches^4^. A recent study demonstrated improved postoperative outcomes for high-risk patients who underwent a total pancreatectomy compared with those who underwent a pancreatoduodenectomy^5^.

Previous research has introduced several CR-POPF prediction models for pancreatoduodenectomy, including the Fistula Risk Score (FRS) and the updated alternative Fistula Risk Score (ua-FRS)^6,7^. These risk models are commonly used but have limitations, including their reliance on subjective intraoperative assessments (for example the texture of the pancreas) and their inability to provide predictions before surgery.

Radiomics is an approach for extracting features from medical images. It enables objective approaches for texture analysis and can uncover new parameters, some invisible to the human eye^8^. Previous studies have investigated the use of computed tomography (CT)-based-radiomics models to predict CR-POPF^9–13^. However, radiomics-based models which are publicly available and externally validated are currently lacking. The objective of this study was to develop and externally validate a publicly available radiomics preoperative-Fistula Risk Score (RAD-FRS) in patients undergoing pancreatoduodenectomy using radiomic features from preoperative CT scans. Such a model could lead to automatic CR-POPF risk prediction in CT reports.

## Methods

The Medical Ethics Review Committee of the Amsterdam University Medical Center (UMC) approved this study protocol and waived the need for informed consent. The Ethics Committee of Verona and Rovigo Provinces approved the utilization of data from the validation cohort (PAD-R, 1101CESC). The study adhered to the STROBE guidelines^14^. All patients were managed per institutional practices. At the Amsterdam UMC, it was standard procedure to insert an abdominal drain after resection, and the routine administration of somatostatin analogues was not performed. The levels of drain fluid amylase were measured on postoperative days 1 and 3. At Verona University Hospital, an assessment of the risk associated with CR-POPF was carried out intraoperatively using the FRS. In instances where a drain was employed, the levels of drain fluid amylase were measured on both postoperative days 1 and day 5.

### Patients

For model design, adult patients after pancreatoduodenectomy in one of the two locations of the Amsterdam UMC (Vrije Universiteit Medical Center) were included from the Dutch Pancreatic Cancer Audit (January 2013–December 2018). Exclusion criteria were patients with a poor quality CT scan or CTs with slice thickness >3.0 mm. A poor quality CT scan was defined as a poor scan due to artefacts (for example respiratory motion artefacts). For external validation of the model, adult patients after pancreatoduodenectomy in the Verona University Hospital were included (January 2020–January 2021). The same eligibility criteria were applied. These data sets will be referred to as the Amsterdam and Verona data sets respectively.

### Data acquisition and outcome

The following patient demographics and tumour characteristics were retrospectively obtained from electronic health records: age (years), sex, BMI (kg/m^2^), ASA classification, pre-existing diabetes, application of neoadjuvant therapy, surgical approach (that is open/laparoscopic/robotic surgery), pancreatic duct size, pancreatic texture, intraoperative blood loss, length of surgical procedure, pathology. The pancreatic duct size was measured intraoperatively with a ruler. The pancreatic texture was assessed subjectively by the surgeon feeling intraoperatively and in minimally invasive surgery, the texture of the resected pancreatoduodenectomy specimen was determined. Contrast-enhanced CT scans were obtained from the picture archiving and communication systems. The primary outcome was a clinically relevant POPF, defined as grade B or C according to the 2016 International Study Group on Pancreatic Surgery (ISGPS) definition^15^.

### Data sets

**Fig. 1:**
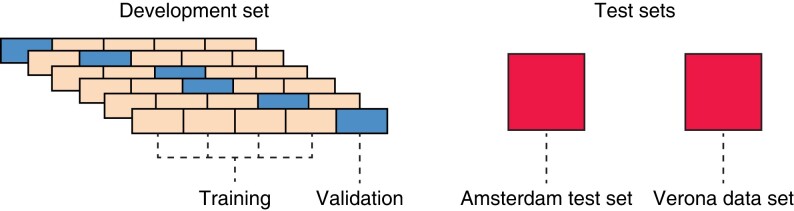
The Amsterdam data set (*n* = 118) was split into the Amsterdam development (90 per cent, *n* = 106) and test set (10 per cent, *n* = 12). The Amsterdam development set was further split into the Amsterdam training and validation set using five-fold cross-validation. The best out of 25 models with regards to the AUROC on the Amsterdam test set was evaluated on the Verona data set (*n* = 57). AUROC, area under the curve—receiver operating characteristic.

Two full data sets (the Amsterdam and Verona data sets) were used. The Amsterdam data set was further divided into three subdata sets as follows. First, the Amsterdam data set was divided into two sets: the Amsterdam development set, comprising 90 per cent of the Amsterdam data set, and the Amsterdam test set, comprising the remaining 10 per cent of the data. Within the Amsterdam development set, there was a further division, creating Amsterdam training and validation sets using five-fold cross-validation. The models were trained on the Amsterdam development set and evaluated on both the Amsterdam test set and the Verona data set. The data splits of the Amsterdam and Verona data sets are visualized in *Fig. 1*.

### Image segmentation and radiomic analysis

We constructed a radiomics workflow consisting of the segmentation of the volume of interest (VOI) and radiomic feature extraction, feature selection and model construction on the Amsterdam development set, and performance evaluation on the Amsterdam test set and Verona data set.

**Fig. 2:**
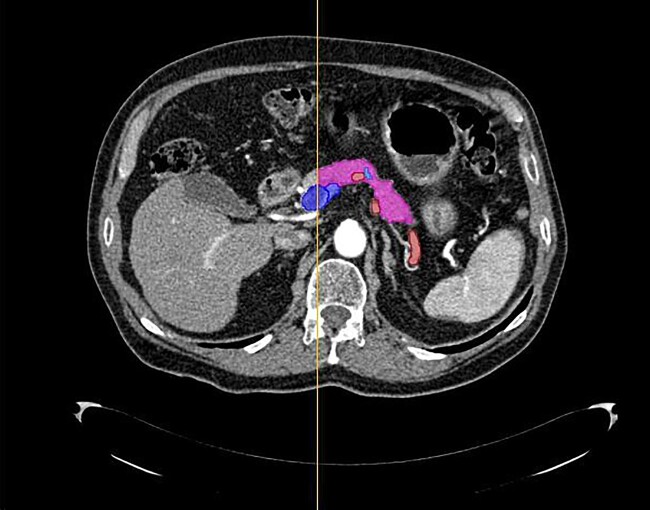
An example of a contrast-enhanced CT scan in the early arterial phase of the abdomen, showing the contoured pancreatic tissue (pink), pancreatic duct (light blue), splenic artery (red) and the superior mesenteric vein (dark blue) in an axial image The vertical yellow line indicates the midline of the superior mesenteric vein, with the pancreas annotated on the left side. The volume of interest consisted of the pancreatic tissue and pancreatic duct. CT, computed tomography.

A PhD candidate specializing in the field of radiology and surgery (E.I.) and a resident in radiology (I.V.) manually pre-segmented the VOI in either the (late) arterial or portal venous phase using 3D slicer version 4.11.20210226 (www.slicer.org)^16^. Subsequently, an experienced abdominal radiologist (Y.N. or F.S. in the Amsterdam data set and R.R. in the Verona data set), who was blinded to the patient’s outcome, finalized the segmentation. The VOI represented the pancreatic remnant, segmented distal/lateral (that is towards the tail) from the midline of the superior mesenteric vein, considering that all patients underwent a pancreatoduodenectomy. The segmentation encompassed both pancreatic tissue and the pancreatic duct. The surrounding vessels (that is splenic artery, splenic vein, superior mesenteric vein and portal vein) were segmented to prevent their inclusion in the pancreatic segmentation mask but were not part of the final VOI (*Fig. 2*). If both the arterial and portal venous phases were available, the pancreas was segmented in the (late) arterial phase. If the arterial phase was unavailable, the pancreas was segmented in the portal venous phase.

Radiomic features were extracted from each VOI of patients in the Amsterdam data set using PyRadiomics version 3.0 (http://github.com/radiomics/pyrdiomics)^17^. Subsequently, 25 Amsterdam development test splits were created for final model selection. For each split, 90 per cent of the Amsterdam data set was allocated to training and 10 per cent to testing. The features of each Amsterdam development and test set were normalized independently using the MinMax scaler. Two feature reduction methods were serially applied to the Amsterdam development sets to select the most predictive features for each split: removing of features with a variance of less than 0.001 and with an importance near zero using least absolute shrinkage and selection operator (LASSO) feature selection.

Four machine-learning (ML) classifiers were fitted to the Amsterdam development sets: support vector machine, logistic regression, k-nearest neighbour and random forest. Before training, the minority classes in the Amsterdam development sets were oversampled with a sampling strategy of 0.9, and subsequently, the majority classes were undersampled. To create more equally sized groups based on the presence or absence of CR-POPF, the data was manually undersampled. Finally, random Gaussian noise with a mean of zero and a variance of 0.2 was added to the Amsterdam development sets. A five-fold cross-validation grid search optimizing area under the curve—receiver operating characteristic (AUROC) was used to find the hyperparameters and fit all four ML classifiers on the Amsterdam development sets. All four ML classifiers were optimized, fitted and evaluated on all 25 Amsterdam development-test splits. The best-performing ML classifier with regards to AUROC on the Amsterdam test set was validated on the Verona data set. The performance of this model on both the Amsterdam test set and Verona data set was evaluated using the AUROC, sensitivity, specificity, positive predictive value (PPV), negative predictive value (NPV) and calibration plots.

### Statistical analysis

Statistical analysis was conducted using Python version 3.7 (Python Software Foundation, Wilmington, DE, USA) and R-Studio version 2022.07.1 (R Studio Team (2018), RStudio: Integrated Development for R. RStudio Inc., Vienna, Austria). The AUROC of the RAD-FRS, FRS and ua-FRS was reported with a 95 per cent confidence interval (95 per cent c.i.). The maximum Youden’s *J* value from the AUROC was used to identify the cut probability where prediction discrimination was maximum according to sensitivity and specificity. The AUROC of the RAD-FRS was compared with the AUROC of the FRS and ua-FRS in the Verona data set using DeLong’s test. Calibration curves were used to compare the observed and estimated probability of the models. A *P* value <0.050 was considered statistically significant. Continuous variables were reported as mean with a standard deviation (s.d.) or median with an interquartile range (i.q.r.) if the distribution was skewed. Dichotomous, ordinal, and nominal variables are presented as numbers and percentages.

## Results

**Fig. 3:**
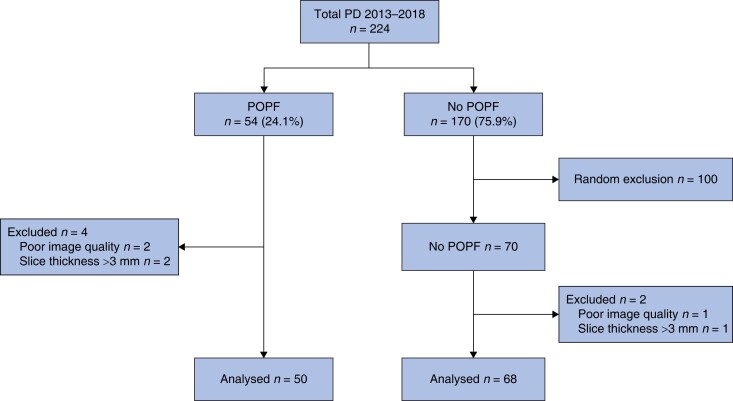
Flow chart of the Amsterdam data set Flow chart showing the selection process of the Amsterdam data set. A total of 100 patients were excluded in the group without CR-POPF to create more equally sized groups based on the presence or absence of CR-POPF. Further exclusion criteria were: poor image quality (*n* = 3) and slice thickness above 3 mm (*n* = 3). A total of 50 patients with CR-POPF and 68 without CR-POPF were found eligible for analysis. PD, pancreatoduodenectomy; CR-POPF, clinically relevant postoperative pancreatic fistula; CT, computed tomography.

**Fig. 4:**
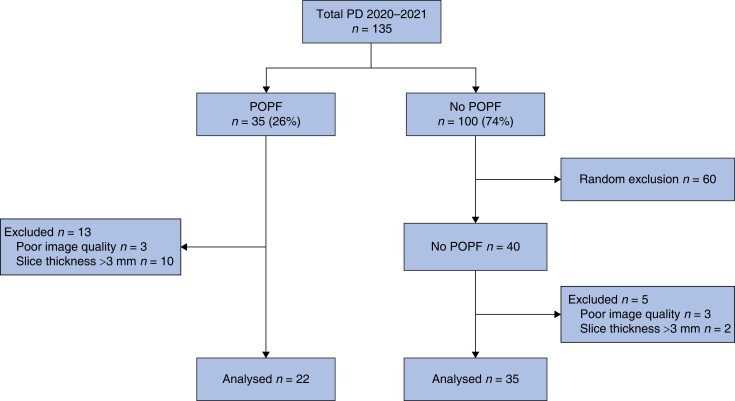
Flow chart of the Verona data set Flow chart showing the selection process of the Verona data set. A total of 60 patients were excluded in the group without CR-POPF to create more equally sized groups based on the presence or absence of CR-POPF. Further exclusion criteria were: poor image quality (*n* = 6) and slice thickness above 3 mm (*n* = 12). A total of 22 patients with CR-POPF and 35 without CR-POPF were found eligible for analysis. PD, pancreatoduodenectomy; CR-POPF, clinically relevant postoperative pancreatic fistula; CT, computed tomography.

Overall, 224 patients underwent a pancreatoduodenectomy at UMC Amsterdam in the Netherlands and 135 at the Verona University Hospital in Italy. A total of 89 (25 per cent) developed CR-POPF. In the Amsterdam data set, a total of 106 patients were excluded: 100 patients without CR-POPF were randomly excluded for the analysis as a result of undersampling, three patients were excluded because of a poor quality CT scan, and three did not have slices ≤3.0 mm, resulting in 118 patients that were included in the Amsterdam data set. Of those, 50 developed CR-POPF (*Fig. 3*). In the Verona data set, 135 patients underwent a pancreatoduodenectomy between January 2020 and January 2021, with CR-POPF occurring in 22 patients (*Fig. 4*). As a result of undersampling, 60 patients without CR-POPF were randomly excluded from the analysis. Another 18 patients were excluded, 6 due to poor CT scan quality, and 12 did not have slices ≤3.0 mm. In total, 57 patients were included in the Verona data set, of whom 22 developed CR-POPF.

### Characteristics of the Amsterdam and Verona data sets

The clinical characteristics of both the Amsterdam and Verona data sets are shown in *Table 1*. The data sets differed in sex (36 per cent female in the Amsterdam data set *versus* 45 per cent in the Verona data set), the application of neoadjuvant therapy (14 per cent in the Amsterdam data set *versus* 58 per cent in the Verona data set), surgical approach (15 per cent of patients in the Amsterdam data set underwent a laparoscopic pancreatoduodenectomy *versus* 0 per cent in the Verona data set), and application of a prophylactic somatostatin analogue (15.3 per cent in the Amsterdam data set *versus* 31.2 per cent in the Verona data set). The Amsterdam data set comprised 118 patients with a median age of 68 years (i.q.r. 59–79 years) and a mean BMI of 25.7 kg/m^2^ (s.d. 3.9 kg/m^2^). The Verona data set included 57 patients with a median age of 68 years (i.q.r. 61–75) and a mean BMI of 25 kg/m^2^ (s.d. 4.1 kg/m^2^). Reconstruction and acquisition parameters used to obtain the CT scans of the Amsterdam and Verona data sets are listed in *Table S1*.

**Table 1:** Baseline characteristics of the Amsterdam and Verona data sets

	Amsterdam data set (*n* = 118)	Verona data set (*n* = 57)
**Age (years), median (i.q.r.)**	68 (59–79)	68 (61–75)
**Sex (female), no. (%)**	43 (36%)	25 (45%)
**BMI (kg/m^2^), mean(s.d.)**	26(3.9)	25(4.1)
**ASA classification, no. (%)**		
I & II	91 (77%)	42 (74%)
III & IV	27 (23%)	15 (26%)
**Diabetes mellitus, no. (%)**	20 (17%)	10 (18%)
**Neoadjuvant therapy, no. (%)**	16 (14%)	33 (58%)
**Preoperative biliary drainage, no. (%)**	35 (29.6%)	17 (29.8%)
**Laparoscopic procedure, no. (%)**	18 (15%)	0
**Pancreatic duct size in mm, median (i.q.r.)**	3 (2–4)	4 (3–5)
**Soft pancreas, no. (%)**	76 (64%)	32 (55%)
**Intraoperative blood loss in ml, median (i.q.r.)**	400 (380–420)	480 (350–792)
**Length of surgical procedure (minutes), median (i.q.r.)**	325 (233–379)	390 (327–465)
**Pathology, no. (%)**		
PDAC	56 (48%)	38 (67%)
Distal cholangiocarcinoma	22 (19%)	3 (5.3%)
Ampullary carcinoma	13 (11%)	3 (5.3%)
Duodenal carcinoma	10 (8.5%)	4 (7.0%)
Cystic neoplasm (IPMN, serous cysts, MCN)	7 (5.9%)	3 (5.3%)
Inflammation/pancreatitis	5 (4.2%)	0
NET	4 (3.4%)	6 (10.5%)
Chronic pancreatitis	1 (0.8%)	0
**Administration of prophylactic somatostatin analogues, no. (%)**	18 (15.3%)	18 (31.2%)

Data are n (%) unless otherwise stated. i.q.r., interquartile range; PDAC, pancreatic ductal adenocarcinoma; IPMN, intraductal papillary mucinous neoplasm; MCN, mucinous cystic neoplasm; NET, neuro-endocrine tumour.

### Amsterdam data set

**Fig. 5:**
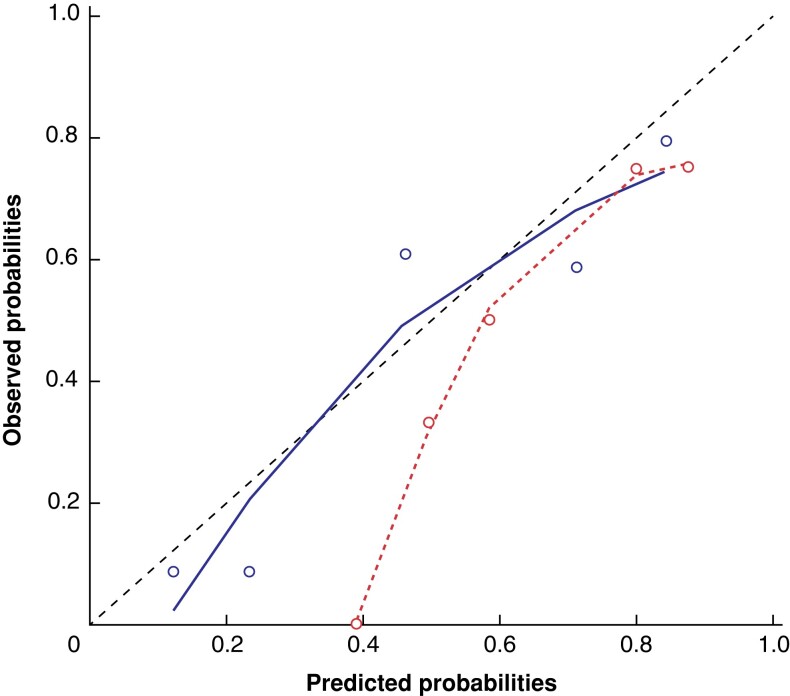
A calibration plot of the RAD-FRS in the Amsterdam test set and Verona data set The black dots represent the quintiles of the observed probabilities by quintiles of the predicted probabilities of the Amsterdam test set, while the white triangles represent the same for the Verona data set. The dashed line represents the ideal performance of the score. RAD-FRS, radiomics preoperative-Fistula Risk Score.

In the Amsterdam data set, 103 (87 per cent) CT scans were segmented in the (late) arterial phase and 15 (13 per cent) in the portal venous phase. A total of 120 radiomic features were extracted. These features are listed in *Table S2*. The final RAD-FRS model included three radiomic features: one grey level dependence matrix feature (grey level non-uniformity) and two 3D shape-based features (voxel volume and minor axis length). The grey level non-uniformity feature quantifies grey level dependencies in an image and measures the variability in intensity values in the image. This feature corresponds to pancreatic texture. The 3D shape-based features include descriptors of the three-dimensional size and shape of the VOI. The voxel volume feature corresponds to the volume of the VOI and thus the remnant of the pancreas volume. The minor axis length yields the second largest axis length of the VOI and could correlate to the pancreatic thickness. The random forest model performed best with an area under the curve (AUC) of 0.90 (95 per cent c.i. 0.71 to 0.99) in the Amsterdam test set (*n* = 12). This model had a sensitivity of 1.00, specificity of 0.67, PPV of 0.71 and NPV of 1.00 in the Amsterdam test set. For the calibration curve of the Amsterdam test set, see *Fig. 5*. The results of the other three machine learning models are listed in *Table 2*.

**Table 2:** Predictive performances of the four machine learning models on the Amsterdam test set

	AUC	Sensitivity	Specificity
**Random forest**	0.90	0.99	0.67
**Logistic regression**	0.86	0.90	0.62
**Support vector machine**	0.81	0.98	0.53
**K-nearest neighbour**	0.80	0.80	0.75

AUROC, area under the curve–receiver operating characteristics.

### Verona data set

In the Verona data set, 52 (91 per cent) CT scans were segmented in the (late) arterial phase and 5 (9.1 per cent) in the portal venous phase. The AUC of the random forest model on the Verona data set (*n* = 57) was 0.81 (95 per cent c.i. 0.69 to 0.92), with a sensitivity of 0.96, specificity of 0.66, PPV of 0.64 and NPV of 0.96. The calibration curve indicated that the model’s performance was consistent with the observed data in the Verona data set and demonstrated an improvement in the consistency between estimated and observed probabilities compared with the Amsterdam test set (*Fig. 5*).

### Comparison with the FRS and ua-FRS

**Fig. 6:**
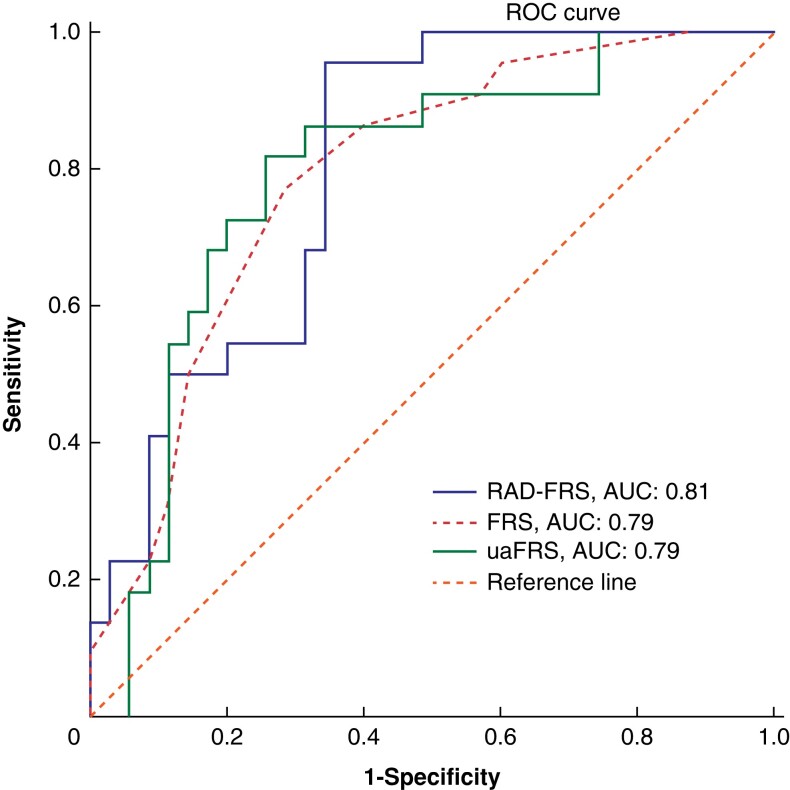
Comparison of the ROC curves for RAD-FRS, ua-FRS and FRS in predicting CR-POPF in the Verona data set The reference line represents the performance of a random guess. ROC, receiver operating characteristics; AUC, area under the curve; RAD-FRS, radiomics preoperative-Fistula Risk Score; ua-FRS, updated alternative Fistula Risk Score; FRS, Fistula Risk Score; CR-POPF, clinically relevant postoperative pancreatic fistula.

The AUC of the RAD-FRS was 0.81 (95 per cent c.i. 0.60 to 0.92) on the Verona data set, which was comparable with that of the FRS (AUC 0.79, 95 per cent c.i. 0.67 to 0.91, DeLong test: *P* = 0.850) and ua-FRS (AUC 0.79, 95 per cent c.i. 0.66 to 0.91, DeLong test: *P* = 0.870) (*Fig. 6*).

## Discussion

The RAD-FRS is the first externally validated and publicly available prediction model for CR-POPF using radiomic features of preoperative CT scans, which performed well with similar external validity to two commonly used risk models for CR-POPF (AUC 0.81), the FRS (AUC 0.79) and ua-FRS (AUC 0.79)^6,7^. Such a radiomics-based model could assist surgeons in counselling patients and making more informed treatment decisions in the preoperative setting. Moreover, accurate risk stratification of CR-POPF in the preoperative setting can assist in determining the best surgical approach for high-risk or frail patients.

Four previous studies used radiomic features from the preoperative CT scan to predict CR-POPF^9–13^. These studies included a comparable number of patients after pancreatoduodenectomy, ranging from 100 to 250. In these studies, the AUC, sensitivity and specificity were also comparable to the present study, with the AUC ranging from 0.76 to 0.81 in the Amsterdam test set (*Table 3*). However, these studies lacked external validation and did not include patients who received neoadjuvant or induction therapy. The present study did include patients with neoadjuvant therapy. However, its use was still relatively limited during the examined study interval, especially in the Amsterdam data set (14 per cent and 58 per cent of all patients and 27 per cent and 75 per cent of patients with pancreatic cancer, in the Amsterdam data set and Verona data set respectively).

**Table 3:** Comparison of the performance of the RAD-FRS model with other published studies that have used radiomic features derived from preoperative CT scans to predict the occurrence of CR-POPF

All risk	AUROC	Sensitivity	Specificity
**RAD-FRS**			
Amsterdam test set	0.90	0.99	0.67
Verona data set	0.81	0.96	0.66
**Capretti *et al*.** ^9^			
Internal test set	0.81	0.82	0.57
External test set	Missing	Missing	Missing
**Kambakamba *et al*.** ^10^			
Internal test set	0.78	0.76	0.64
External test set	Missing	Missing	Missing
**Lin *et al*.** ^11^			
Internal test set	0.79	0.63	0.78
External test set	Missing	Missing	Missing
**Zhang *et al*.** ^12^			
Internal test set	0.76	Missing	Missing
External test set	Missing	Missing	Missing

CR-POPF, clinically relevant postoperative pancreatic fistula; CT, computed tomography; RAD-FRS, radiomics preoperative-Fistula Risk Score; AUROC, area under the curve–receiver operating characteristics.

Although the RAD-FRS has the advantage over the FRS and ua-FRS in that it predicts CR-POPF before surgery, it is currently less convenient to use than the FRS and ua-FRS due to the need for time-consuming manual segmentation of the pancreatic parenchyma. To make the RAD-FRS clinically applicable, auto-segmentation is required. Future work will involve developing an auto-segmentation model using deep learning techniques to fully automate the segmentation of the pancreatic parenchyma. Such a fully automated CR-POPF prediction model has the potential to be integrated into commonly used hospital radiology systems and CT scanners allowing for predictions at scanning time. These risk assessments could facilitate personalized treatment decisions by aiding surgeons in deciding on mitigation strategies and whether to proceed with a pancreatoduodenectomy. The initial results of a fully automatic CR-POPF prediction model developed by our group are promising. Another area of future work should involve the combination of radiomic features with preoperative clinical data, as demonstrated in a model developed by Lin *et al*., which exhibited excellent predictive performance^11^. Bhasker *et al*. also incorporated mesh-based volume to this combination model and demonstrated a significant improvement of the predictive performance with this addition^13^. However, both the combination models developed by Lin *et al*. and Bhasker *et al*. require external validation and are currently not publicly available.

Several limitations of this study should be addressed. First, patients included in this study were managed following institutional practices. Therefore, differences in the perioperative management, including drain management and the administration of somatostatin analogue, between both centres were conceivable. Despite these potential differences, the RAD-FRS performed well on the Verona data set, showing its potential across heterogeneous populations. Second, this is a retrospective study with all its inherent limitations. A prospective study is needed to investigate the true value of the tool and to assess whether it can influence clinical decision-making. Third, most patients in both data sets underwent open surgery, and a subgroup analysis of open and minimally invasive pancreatic surgery is therefore lacking. As minimally invasive pancreatoduodenectomy is more commonly performed, future studies will need to validate the usefulness of the model in such patients for future applications^18^. Fourth, manual undersampling was used to create more equally sized groups based on the presence or absence of CR-POPF. However, manually annotating patients without CR-POPF would be futile, considering that undersampling would be one of the first preprocessing steps. Fifth, we did report calibration, but for clinical use, this would require correction for the undersampling. The discriminative ability of the RAD-FRS was promising as evaluated on the Verona data set. Future work should enable absolute risk estimations in the total population.

The RAD-FRS can potentially improve patient prognosis and could eventually assist surgeons in making tailored treatment decisions. The externally validated RAD-RFS model and the code used for training, validation and evaluation are provided at www.pancreascalculator.com and www.github.com/PHAIR-Consortium/POPF-predictor.

## Supplementary Material

zrad100_Supplementary_Data

## Data Availability

The externally validated RAD-RFS and the code used for training, validation and evaluation are provided at www.pancreascalculator.com and www.github.com/PHAIR-Consortium/POPF-predictor.
