# Supplementary material for: Radiomics preoperative-Fistula Risk Score (RAD-FRS) for pancreatoduodenectomy: development and external validation
Source: BJS Open. 2023 Oct 9;7(5):zrad100. doi: 10.1093/bjsopen/zrad100 (PMC10561131; doi:10.1093/bjsopen/zrad100)
Supplement: zrad100_Supplementary_Data [file zrad100_supplementary_data.docx]

**Title**

Radiomics preoperative-Fistula Risk Score (RAD-FRS) for pancreatoduodenectomy: development and external validation

Erik W. Ingwersen MD^1,2,3*^, Jacqueline I. Bereska MSc^2,4,5*^, Alberto Balduzzi MD^6^, Boris V. Janssen BSc^2,3,7^, Marc G. Besselink MD PhD^2,3,7^, Geert Kazemier MD PhD^1,2^, Giovanni Marchegiani MD PhD^6^, Giuseppe Malleo MD PhD^6^, Henk A. Marquering PhD^4,5^, C. Yung Nio MD^4^, Riccardo de Robertis MD^8^, Roberto Salvia MD PhD^6^, Ewout W. Steyerberg PhD^9^, Jaap Stoker MD PhD^2,3,4^, Femke Struik MD^4^, Inez M. Verpalen MD PhD^4#^, Freek Daams MD PhD^1,2#^, for the Pancreatobiliary and Hepatic Artificial Intelligence Research (PHAIR) consortium

^1^Amsterdam UMC, location Vrije Universiteit Amsterdam, Department of Surgery, Amsterdam, The Netherlands ^2^Cancer Center Amsterdam, the Netherlands
^3^Amsterdam Gastroenterology Endocrinology and Metabolism, the Netherlands ^4^Amsterdam UMC, location University of Amsterdam, Department of Radiology and Nuclear Medicine, Amsterdam, The Netherlands ^5^Department of Biomedical Engineering and Physics department, University of Amsterdam, The Netherlands ^6^Department of Surgery and Oncology, Unit of General and Pancreatic Surgery, University of Verona Hospital Trust, Verona, Italy ^7^Amsterdam UMC, location University of Amsterdam, Department of Surgery, Amsterdam, the Netherlands ^8^University Hospital G.B. Rossi, University of Verona, Department of Radiology, Verona, Italy ^9^Department of Biomedical Data sciences, Leiden University Medical Center, Leiden, The Netherlands
*this authors contributed equally to this work #shared last authorship

**Corresponding author**

F. Daams, MD, PhD
Amsterdam UMC, location Vrije Universiteit Amsterdam

Department of Surgery, De Boelelaan 1117, Amsterdam, the Netherlands.

Telephone: +31 6 55 68 85 42. Email: [f.daams@amsterdamUMC.nl](mailto:f.daams@amsterdamUMC.nl)

ORCID ID: 0000-0001-6323-6342

**Supplementary Materials - Index**

| **Supplementary Figures and Tables** |  |
| --- | --- |
| Supplementary Table 1 | *page 2* |
| Supplementary Table 2 | *page 3* |
|  |  |

**Supplementary Table 1. Acquisition and reconstruction parameters of the CT scans of the Amsterdam and Verona datasets.**

| **Parameter** | Amsterdam dataset | | Verona test set |
| --- | --- | --- | --- |
| Pitch | \| 0.6 – 1.388 \| \| --- \| | 0.5 – 0.891 | |
| X-ray tube current | 17 – 183 mA | 200 – 250mA | |
| kVp | 90 – 120 kVp | 90 – 120 kVp | |
| Tube rotation speed | 0.4 – 0.7 ms | 0.4 – 0.75 ms | |
| Slice thickness | 1.0 – 3.0mm | 1.0 – 2.0 mm | |
| Width | 400 - 934 | 300 – 400 | |
| Height | 300 - 975 | 300 - 400 | |
| Machine manufacturer | SIEMENS, GE Healthcare | | Philips Brilliance 64  Siemens Somatom Definition Edge 128 |

Supplementary Table 1. Upper value is the maximum and lower value is the minimum of all values present in the dataset for that specific parameter.

CT; computed tomography, kVp; kilovoltage peak

**Supplementary Table 2. List of radiomic features extracted from Pyradiomics.**

| *Feature class* | *Number of features* |
| --- | --- |
| First order statistics | 19 features |
| Shape-based (3D) | 16 features |
| Shape-based (2D) | 10 features |
| Gray Level Co-occurrence Matrix | 24 features |
| Gray Level Run Length Matrix | 16 features |
| Gray Level Size Zone Matrix | 16 features |
| Neighboring Gray Tone Difference Matrix | 5 features |
| Gray Level Dependence Matrix | 14 features |

Supplementary Table 2 lists all the extracted radiomic features. These features are extracted using Pyradiomics. A description of each feature can be found on: [https://pyradiomics.readthedocs.io/en/latest/features.html](https://eur04.safelinks.protection.outlook.com/?url=https%3A%2F%2Fpyradiomics.readthedocs.io%2Fen%2Flatest%2Ffeatures.html&data=05%7C01%7Ce.ingwersen%40amsterdamumc.nl%7Ccf0af47c0f2f4805ae5808db8081175a%7C68dfab1a11bb4cc6beb528d756984fb6%7C0%7C0%7C638245064285226232%7CUnknown%7CTWFpbGZsb3d8eyJWIjoiMC4wLjAwMDAiLCJQIjoiV2luMzIiLCJBTiI6Ik1haWwiLCJXVCI6Mn0%3D%7C3000%7C%7C%7C&sdata=G33zZXW5Na6B6TmvmDNFMmGqS7DCG5wWDaF4W0JIJkY%3D&reserved=0)
